# Constructing the Indicators of Assessing Human Vulnerability to Industrial Chemical Accidents: A Consensus-based Fuzzy Delphi and Fuzzy AHP Approach

**DOI:** 10.1371/currents.dis.526884afe308f8876dce69c545357ecd

**Published:** 2017-04-10

**Authors:** Farin Fatemi, Ali Ardalan, Benigno Aguirre, Nabiollah Mansouri, Iraj Mohammadfam

**Affiliations:** Department of Disaster Public Health, School of Public Health, Tehran University of Medical Sciences, Tehran, Iran; Department of Disaster Public Health, Head of Disaster Public Health Department, School of Public Health, Tehran University Medical of Sciences, Tehran, Iran; Disaster Research Center, University of Delaware, Delaware, United States; Department of Health, Safety, and Environmental Management, Science and Research Branch of Islamic Azad University, Tehran, Iran; Department of Occupational Health, School of Public Health, Hamadan University of Medical Sciences, Hamadan, Iran

## Abstract

**Introduction:**

Industrial chemical accidents have been increased in developing countries. Assessing the human vulnerability in the residents of industrial areas is necessary for reducing the injuries and causalities of chemical hazards. The aim of this study was to explore the key indicators for the assessment of human vulnerability in the residents living near chemical installations.

**Methods:**

The indicators were established in the present study based on the Fuzzy Delphi method (FDM) and Fuzzy Analytic Hierarchy Process (FAHP). The reliability of FDM and FAHP was calculated. The indicators of human vulnerability were explored in two sets of social and physical domains. Thirty-five relevant experts participated in this study during March-July 2015.

**Results:**

According to experts, the top three indicators of human vulnerability according to the FDM and FAHP were vulnerable groups, population density, and awareness. Detailed sub-vulnerable groups and awareness were developed based on age, chronic or severe diseases, disability, first responders, and residents, respectively. Each indicator and sub-indicator was weighted and ranked and had an acceptable consistency ratio.

**Conclusions:**

The importance of social vulnerability indicators are about 7 times more than physical vulnerability indicators. Among the extracted indicators, vulnerable groups had the highest weight and the greatest impact on human vulnerability. however, further research is needed to investigate the applicability of established indicators and generalizability of the results to other studies.

**Key words:**

Fuzzy Delphi; Fuzzy AHP; Human vulnerability; Chemical hazards

## Introduction

Technological advances, and discovery and consumption of thousands of chemicals have met many of the human needs at industrial and economical levels. On the other hand, in some cases, these chemicals have resulted in catastrophic consequences such as explosion, fire, leak, and release of chemicals^1^. In this situation, there is a high potential for damaging economic, environmental, and human resources and concern is growing about the public health effects, as was the case at Flixbrough, England, Bhopal, India, and Seveso, Italy^2^^,^^3^^,^^4^.

The trend of industrialization has been growing quickly on the basis of market demands in Iran and industrial chemical accidents have increased in the recent years^5^. Therefore, a comprehensive chemical safety program should be a priority of the authorities in the areas that are prone to chemical accidents^6^. Identifying the human vulnerability can be part of the mentioned program. Human vulnerability can be defined as the factors that make people working in the chemical installation or living in the proximity of such places vulnerable to harms and injuries^2^. Assessment of human vulnerability to chemical accidents through identifying the relevant indicators could be the first step towards reducing the chemical risk and establishing appropriate mitigation programs^7^. Most of the available studies have focused on defining the indicators of human vulnerability in natural disasters^8^. However, a challenging area in this field is whether they can be used in man-made disasters such as chemical releases.

The Delphi method is usually recommended for selecting indicators in many fields based on the mean value of expert opinions^9^. The Fuzzy theory has been also suggested for the analysis of such studies in order to prevent expert judgment from the influence of extreme values, as Ishikawa did in 1993^10^. Applying the Fuzzy Delphi Method (FDM) has the ability to combine the participants’ opinions more reasonably^11^. After finalizing the indicators, the Fuzzy Analytic Hierarchy Process (FAHP) approach was used to prioritize the relevant indicators under a hierarchy model^12^. The combination of FDM and FAHP can calculate weight values and enhance objectivity and accuracy of indicators and their relevant variables^13^.

The main goal of this study was to construct and prioritize the indicators of human vulnerability in chemical accidents through applying a Fuzzy Delphi-AHP approach. The identification and prioritization indicators of social and physical vulnerability presented in this paper may improve the assessment of human vulnerability in chemical accidents. In addition, they will provide a new insight for the experts of disaster management planning and strategy department of chemical installations.

**Figure:**
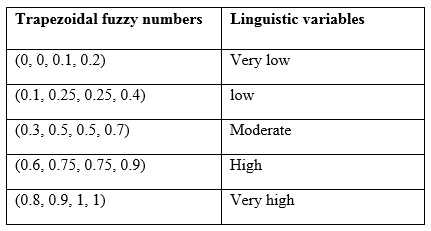
**Table 1:** Linguistic variables and relevant trapezoidal fuzzy numbers in this study

**Results**

This study was conducted in 3 rounds. A total of 42 experts, including Iranian and American experts with different ages and years of experience in disaster, safety, crisis management, and chemical and social sciences were invited to participate in the Delphi study.

The response rates were 76.2%, 90.6% and 96.5% in the 1st, 2nd, and 3rd round of the Delphi study, respectively.

## Methods

This study used a Fuzzy Delphi-AHP method to identify the potential indicators of human vulnerability in chemical accidents in 2015. This method enables the researchers to reach an expert consensus about the topic during multiple rounds.

In order to simplify the survey process, two primary vulnerability indicators, including social and physical indicators, were proposed considering a review of the literature and focus group discussion^2^. Based on two primary vulnerability indicators, a questionnaire with a five point Likert scale from ‘‘very low” to ‘‘very important” was designed. Because of having few experts from other countries and also the physical distance among Iranian experts, the online Delphi method was appropriate as it does not require the physical presence of experts^14^. This study was conducted in 3 sequential rounds of a group of experts, who are anonymous to each other but the selecting the experts was purposeful. The including criteria for Participating the experts to this study were:

- Having the academic degree (PhD, MSc) in one of the mentioned majors: Health Safety Environment (HSE), Chemistry Engineering in Crisis Management branch, Health in Disasters and Emergencies, Social Sciences and Epidemiology.
- Working as the faculties (University or Research Institutes), PhD students in one mentioned majors or HSE managers in industries.
- Having the empirical studies and relevant peer-reviewed articles in relevant subjects to chemical accident modeling, public safety, disasters and emergencies management.

Also, we used anonymous questionnaires for each round and the analyzer was completely blind to the expert who completed the questionnaire. This implementation method of analysis avoided situations in which the analyzer is dominated by the views of a few.

At each round, all experts were contacted via e-mail to define the importance of the indicators. The participants were given 4 weeks to answer. During this period, 3 reminder emails were sent to the experts to complete the survey in each round. If they did not complete the questionnaire after 4 weeks, they were excluded from the study for the next round. The results of each round were analyzed by the Fuzzy method and Fuzzy Trapezoidal Number (Table 1). The threshold value in each round was 0.7 and the cut point for continuing the rounds of Delphi was 0.2. During the 1st and 2nd rounds, the changes were made in the questionnaire based on the group views. After the 3rd round, the mean value of group responses were calculated for each indicator and fed back to the panelists who could reconsider their views based on the reported mean values of indicators. Then the difference between before and after the reconsideration were calculated. Because the difference value was less than 0.2, the study ended up at this round. Ethical approval was obtained from Ethics Committee of Tehran University of Medical Sciences for the whole project as a PhD dissertation. In this part of work, at the beginning of Delphi the oral consents were provided to participate in the study from all experts. The oral consents were not documented but if they rejected, the Delphi questionnaire would not email for them. Also, they were completely free to reply or not to the emails in the next rounds of Delphi study.

Then, we prioritized the extracted indicators of human vulnerability under a hierarchy model identified by the Fuzzy Analytic Hierarchy Process (FAHP) approach. Also, three matrixes were sent to 8 experts to weight. Then, we entered each calculated weight of the criteria into a separate matrix in a software called ‘Super Decision’ for the application of FAHP and computed the weights of relative indicators to human vulnerability.

## Results

This study was conducted in 3 rounds. A total of 42 experts, including Iranian and American experts with different ages and years of experience in disaster, safety, crisis management, and chemical and social sciences were invited to participate in the Delphi study.

The response rates were 76.2%, 90.6% and 96.5% in the 1st, 2nd, and 3rd round of the Delphi study, respectively.

**A fuzzy trapezoidal number:**
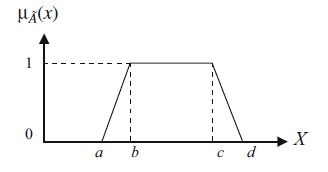

**Figure:**
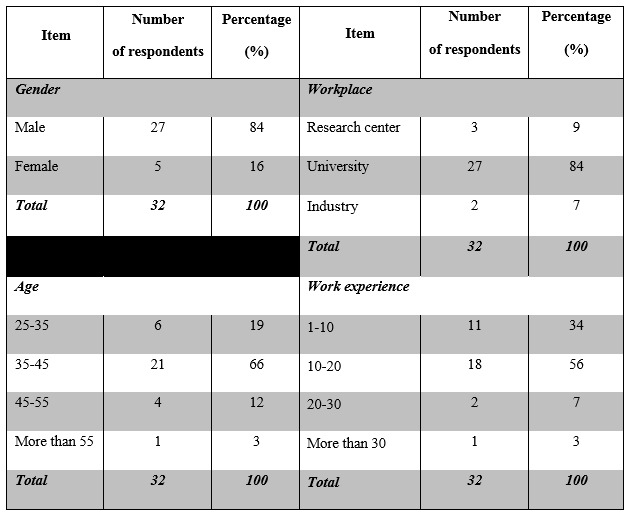
**Table 2:** Descriptive statistics of Delphi participants

First, we extracted 38 indicators of human vulnerability in social and physical domains from a review of the literature. Then, the draft of indicators was emailed for Delphi. Seventeen indicators were deleted and 21 indicators were retained in the first round, including five highest defuzzified values which were population density, public awareness, number of chemical plants and warehouses in the area under study, percentage of equipped health facilities for chemical victim management, and level of safety in chemicals plants. The indicators were screened based on the threshold (The indicators that were greater than 0.7 were retained, and the indicators below 0.7 were deleted. It was found that defuzzified values for the indicators below 0.7 were population growth, minority, migration, dependency, employment and working population in primary, secondary, and tertiary sectors. Also, 11 new indicators were added according the expert views. These indicators were the number of active organizations during disasters and crises in the area, distance between chemical installations and the nearest residential areas, type of communication systems during time of disasters and emergencies, accessibility to location accident, and other supportive systems like fire brigade, health service, ambulance and etc. in factories and companies of the region.

Defuzzified values showed that all 32 indicators were greater than 0.7 and were therefore retained, and no indicator was deleted in the 2nd round. The five greatest values of the indicators belonged to the percentage of chemical plants or warehouses that produced, used, or stored hazardous chemical materials in the area under study, early warning systems, rate of the preparedness of health facilities for chemical accidents, health workers trained in chemical hazards and management of the injuries, and public awareness (0.91, 0.86, 0.84, 0.83, and 0.82, respectively). In the 3rd round, all the mean values of the indicators were greater than 0.7 and retained in the study. The five indicators with the highest averages in the 3rd round were similar to the previous round. Then the calculated mean values of the indicators have been sent to the experts and they were asked to change the importance of the indicators if they wished to. If, after revision, a difuzzified value was more than 0.2, Delphi study was going to continue to the next round. In this study, the calculated revision value was 1.8 and therefore, the study was finished in this round.

Then, we prioritized the 32 factors under a hierarchy model identified by the Fuzzy Analytic Hierarchy Process (FAHP) approach. The analytic hierarchy process model for the assessment of human vulnerability has been demonstrated in Fig. 2.

Then, we prioritized the 32 factors under a hierarchy model identified by the Fuzzy Analytic Hierarchy Process (FAHP) approach. The analytic hierarchy process model for the assessment of human vulnerability has been demonstrated in Fig. 2.

**Analytic Hierarchy Process Mode for Assessment of Human Vulnerability:**
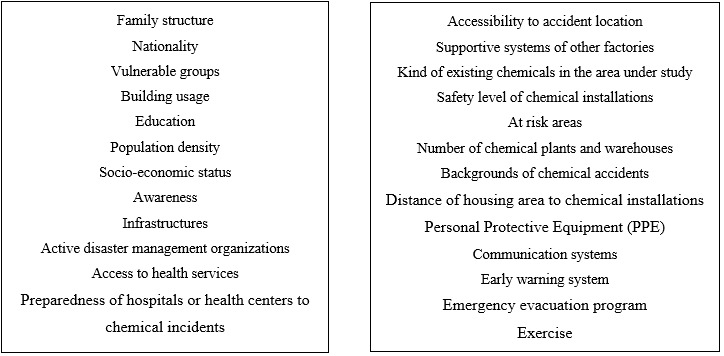

In this study, all calculated consistency ratios from AHP matrixes ranged from 0.00 to 0.09 and fell within the acceptable level of 0.1 as recommended by Satty^12^, indicating that the study participants assigned their weights consistently after examining the priority of the indicators of human vulnerability to industrial chemical accidents.To determine the prioritization of the social and physical vulnerability with regard to the goal of assessing human vulnerability, the weights of determined indicators were calculated and demonstrated in radiated diagrams (Fig. 3 and Fig. 4).

**Radiated diagram of weight of social vulnerability:**
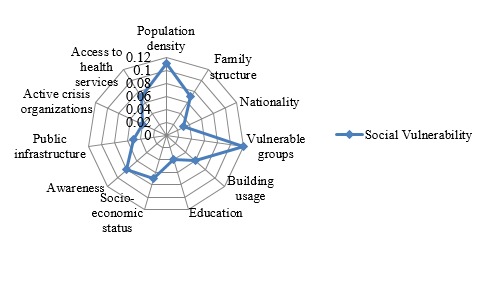

**Radiated diagram of weight of physical vulnerability:**
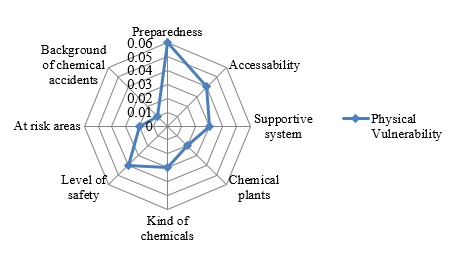

**Figure:**
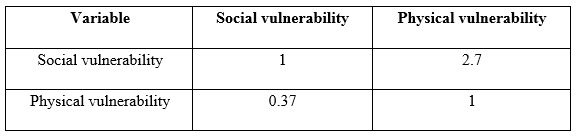
**Table 3:** Weight of indicators to the human vulnerability in chemical accidents

**Figure:**
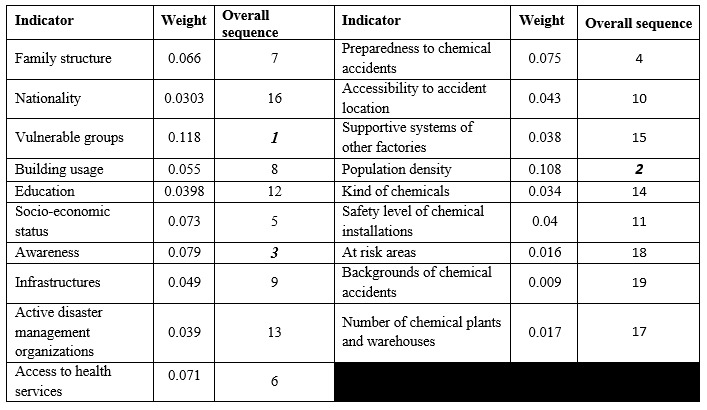
**Table 4:** Weight of indicators to the human vulnerability in chemical accidents

## Discussion

The research findings showed that social vulnerability is 2.7 times more than physical vulnerability in assessing the human vulnerability to chemical accidents in the residential areas. The indicators such as vulnerable groups, population density which is defined as the number of people per square meter in the area under study and public awareness of chemical plants, related hazards, type of chemicals and precautionary measures at time of chemical accidents and emergencies, are the top three indicators in the social vulnerability which was also the highest among the overall weight, while the preparedness of region to chemical accidents, accessibility to the accident location and level of safety in chemical plants and warehouses are the three major indicators in the physical vulnerability.

The “vulnerable groups” possessed the highest weight values (0.118) in the human vulnerability indicators to chemical accidents. This indicator had 4 sub-indicators, including households that had children under 7 years old, member(s) above 65 years old, individual(s) with chronic or severe disease(s), and member(s) with mental or physical disability. In the previous studies about social vulnerability, 1 or 2 of the above conditions were considered as one of the indicators of vulnerability^15^^,^^16^^,^^17^^,^^18^ but four different situations were determined for finding vulnerable households at the same time in this study. Population density can be effective on organizing all facilities required for chemical hazards preparedness and reduction of the relevant vulnerabilities in at risk areas^19^. Training the first responders who attend at the scene of a chemical accident and also increasing the awareness of residents are essential in the industrial areas^20^. The people who live in the proximity of the chemical plants and warehouses should have information about chemicals and their relevant hazards in the vicinity and also, the protective measures they can take if a chemical incident occurs. For instance, many lives would have been saved if people stayed indoors with a wet piece of cloth on their face as a simple safety measure in the Bhopal accident^7^.

Among the indicators of physical vulnerability, “preparedness of the region for chemical hazards” held the highest weight (0.075). This indicator had 7 sub-indicators, including emergency evacuation plan, disaster shelters, percentage of equipped health facilities for chemical hazards management, early warning systems, modern communication instruments, periodic chemical exercises, and providing suitable Personal Protective Equipment (PPE) for residents living near chemical installations. This finding shows that we need to develop required infrastructures for chemical emergency management in the areas which are exposed to chemical hazards. Moreover, the authorities should engage the private sectors which may have resources to contribute to local chemical emergency planning^7^^,^^21^. This result is consistent with the findings of a study by Guizhen^22^. Accessibility to the accident location was another important factor among physical vulnerability indicators. The sub categories of this factor including the distance between the accident location to the nearest main road and traffic and conditions also had a high weight in another study exploring physical vulnerability against fire hazards in Daka, Bangladesh^23^. The level of safety in the chemical installations was the third considerable indicator in physical vulnerability with a weight of 0.04. Chemical regulations and relevant standards should be monitored and enforced in order to promote safety to an acceptable level in chemical plants and warehouses^24^. This strategy has been implemented in the European Union and the countries have succeeded in developing a public health surveillance system for chemical incidents^25^.

## Conclusions

The main contribution of this paper was to identify a draft of effective indicators for assessing human vulnerability to a chemical accident. This study explored the application of the Fuzzy Delphi-AHP Method to quantify the expert views to human vulnerability of the residents living near chemical plants and warehouses. Also, the other possible factors affecting human vulnerability to industrial chemical accidents were considered and two sets of indicators in social and physical domains were constructed. Then, human vulnerability indicators were selected and weighted using the Fuzzy Delphi and Fuzzy AHP method, respectively. The results of the FAHP method indicated that age under 70 or over 65, chronic/severe disease of mental/physical disability (vulnerable groups) was the main risk factor for the human vulnerability of households. In addition, population density and public awareness of chemicals, hazards and precautionary measures in at chemical risk areas were identified as potentially having a greater effect with respect to adverse outcomes of a chemical spill compared to other finalized indicators.

This paper provides implications for both scholars and policy makers to extend their research in man-made disasters with focus on chemical hazards. Previous studies have investigated Social Vulnerability Indicators (SVI) only in natural hazards. However, we added the indicators of physical vulnerability and integrated them with SVI to measure and mapping the human vulnerability in the areas that are prone to chemical accidents. Now, the authorities of region have accessibility to the map of more vulnerable areas in the area under that are more informative for practical purposes such as allocating the needed resources to critical points.

More studies are required to investigate the applicability of the indicators of human vulnerability developed in this paper in different disasters and emergencies. Researchers also need to validate the criteria for generalizability of the results to other studies.

## Limitations

Only English articles were included in the literature review of this article.

## Corresponding Author

Ali Ardalan. Department of Disaster Public Health, School of Public Health, Tehran University of Medical Sciences, Tehran, Iran. Email: aardalan@tums.ac.ir

## Authors Contributions

Farin Fatemi, Dr. Ali Ardalan and Prof. Benigno Aguirre have contributed in study design, acquisition of data, data analysis, drafting the article, and development of the final manuscript. DR. Nabiollah Mansouri and Dr. Iraj Mohammadfam have critically evaluated the draft article. All authors reviewed and approved the final version of the manuscript.

## Data Availability

All relevant data are within the manuscript and the public repository Figshare. Please view data at the following URL: 10.6084/m9.figshare.4732921. For more information, please contact to the author Farin Fatemi (farin.fatemi@gmail.com).

## Competing Interest

The authors have declared that no competing interests exist.
